# Effect of Pre-Existent Sarcopenia on Oncological Outcome of Advanced Thyroid Cancer Patients Treated with Tyrosine Kinase Inhibitors

**DOI:** 10.3390/cancers14194569

**Published:** 2022-09-21

**Authors:** Cristina Dalmiglio, Lucia Brilli, Cristina Ciuoli, Fabio Maino, Laura Valerio, Ida Sannino, Alessandra Cartocci, Susanna Guerrini, Matteo Zanoni, Giuseppe Marrazzo, Maria Antonietta Mazzei, Maria Grazia Castagna

**Affiliations:** 1 Unit of Endocrinology, Department of Medical, Surgical and Neurological Sciences, University of Siena, 53100 Siena, Italy; 2 Department of Medical Biotechnologies, University of Siena, 53100 Siena, Italy; 3 Unit of Diagnostic Imaging, Department of Medical, Surgical and Neuro Sciences and of Radiological Sciences, University of Siena, 53100 Siena, Italy

**Keywords:** sarcopenia, Skeletal Muscle Index, thyroid cancer, tyrosine kinase inhibitors

## Abstract

**Simple Summary:**

Data regarding the effect of pre-existent sarcopenia on the oncological outcome of advanced thyroid cancer patients treated with tyrosine kinase (TKI) are still lacking. The aim of the study was to investigate the prevalence of pre-treatment sarcopenia in Caucasian patients affected by advanced thyroid carcinoma and the impact of this condition on the response to TKIs treatment. Pre-treatment sarcopenia was found in 20.7% of patients, with an increase of up to 38.5% after 12 months of TKI therapy. Pre-treatment sarcopenia significantly affected treatment outcome, emerging as the parameter that has the greatest impact on Progression Free Survival. Sarcopenia might be used as a prognostic factor of TKI treatment outcome and the prevention of this condition, ideally before starting anticancer treatment, could be the strategy to obtain a better efficacy of therapy.

**Abstract:**

(1) Background: Sarcopenia is associated with poor survival and treatment outcomes in several human cancers. The aim of the study was to investigate the prevalence of sarcopenia in a cohort of 58 Caucasian patients with advanced thyroid cancer before and during TKI treatment. The impact of this condition on the outcome of patients was also evaluated. (2) Methods: Sarcopenia was evaluated using the Skeletal Muscle Index (SMI). (3) Results: Pre-treatment sarcopenia was found in 20.7% of patients and this condition significantly affected treatment outcome, emerging as the parameter that has the greatest impact on Progression Free Survival (PFS) (HR 4.29; 95% CI, 1.21–15.11, *p* = 0.02). A significant reduction in SMI values was observed 3 (*p* = 0.002) and 12 months (*p* < 0.0001) after TKI treatment. At a 12-month follow-up, sarcopenia prevalence increased up to 38.5%. Here, 12-month sarcopenia was predicted by a lower SMI (*p* = 0.029), BMI (*p* = 0.02) and weight (*p* = 0.04) and by the presence of bone metastases (*p* = 0.02). (4) Conclusions: This is the first study that evaluated sarcopenia prevalence and its change over time in Caucasian patients with advanced thyroid cancer under TKI therapy. Sarcopenia seems to be a prognostic factor of TKI treatment outcome, suggesting the importance of the assessment of the nutritional status and body composition in advanced thyroid cancer patients.

## 1. Introduction

Sarcopenia, malnutrition, and cancer cachexia often co-exist in patients with advanced cancer and are associated with poorer response to cancer therapy and with reduced survival [1,2,3,4,5]. It is important to recognize the differences between malnutrition, sarcopenia, and cancer cachexia since features overlap between these conditions. Malnutrition is commonly defined as a deficiency of energy intake, which can lead to altered body composition, poor clinical outcomes and impaired physical and mental functioning in patients with chronic diseases [6]. Cachexia has been defined as a complex metabolic syndrome, characterized by loss of muscle with or without loss of fat mass and associated with underlying disease [7]. Hence, the majority of cachectic individuals are also sarcopenic, but most sarcopenic individuals are not considered cachectic. Sarcopenia is one of the elements of the proposed definition for cachexia [8]. Recently, The European Society for Clinical Nutrition and Metabolism (ESPEN) published a consensus paper expanding this definition of cachexia and identifying relevant issues on how to differentiate cachexia and sarcopenia [9]. Sarcopenia is a syndrome characterized by a progressive and generalized loss of skeletal muscle mass and strength, with adverse outcomes including physical disability, poor quality of life, and death [10]. 

Sarcopenia is associated with poor survival and treatment outcomes of anticancer drugs in several human cancers [3,11,12,13]. According to the European Working Group on Sarcopenia in Older People (EWGSOP2), sarcopenia is confirmed by the presence of reduced quantity or quality of muscle mass [14]. Computed Tomography (CT) and Magnetic Resonance (MR) are considered the gold standard for muscle mass measurement because they allow a precise analysis of the anatomical tissues. The area of the appendicular muscles (Skeletal Muscle, SM) at the third lumbar vertebra (L3) level, detectable by CT, significantly correlates with the muscles of the whole body in healthy adults [15]. Skeletal Muscle Index (SMI), obtained by SM normalized by height squared, is one of the recognized methods to estimate sarcopenia. Currently, however, there is still no consensus regarding SMI cut-offs to define sarcopenia. 

Cancer patients have an increased risk of developing sarcopenia. In a recent review, regarding 35 studies and 6894 oncological patients, the overall sarcopenia prevalence before treatment was 38.6%, measured by different methods such as CT, MR, Dual-Energy X-ray Absorptiometry (DXA) or Body Impedance Assessment (BIA). Sarcopenia was associated with a worse Overall Survival (OS) and Progression Free Survival (PFS) and its presence was also associated with a higher rate of post-surgical complications, toxicity induced by chemotherapy, and dose-limiting toxicity (DLT) [16]. In a recent meta-analysis, Rinninella et al. [17] analyzed the prognostic value of sarcopenia in cancer patients undergoing treatment with TKI, showing that, in these patients, a reduced muscle mass had a significant impact on DLT (OR 2.40, IC 95% 1.26–4.58, *p* = 0.008, I2 = 51%).

Up to now, few data regarding sarcopenia are available among patients with advanced thyroid cancer [18,19,20,21]. In two recent Japanese studies, the presence of pre-treatment sarcopenia, measured by SMI, led to poor outcomes in patients with advanced thyroid cancer [20,21]. 

Since data regarding the role of sarcopenia in advanced thyroid cancer patients have been reported only in the Asian population, the main aims of this study were (1) to investigate the prevalence of sarcopenia before and during TKI treatment in a cohort of 58 Caucasian patients with advanced thyroid cancer; (2) to evaluate the impact of sarcopenia on response to TKI treatment in these patients; (3) to identify possible prognostic factors for developing sarcopenia during TKI therapy.

## 2. Materials and Methods

### 2.1. Study Population

We retrospectively evaluated 58 patients with differentiated (papillary, follicular, Hurthle cells) (DTC), poorly differentiated (PDTC), and medullary thyroid cancer (MTC) locally advanced or metastatic, treated with at least one TKI between November 2004 and November 2021. The data collected included: age at diagnosis, gender, anthropometric parameters, ECOG performance status, histological findings, stage at diagnosis, numbers of anatomical sites involved, information on treatment with TKIs (type of TKI, time lapse between diagnosis and treatment start, duration of treatment, reason for discontinuation), tumor response, and data on last follow-up/death. 

### 2.2. Assessments and Definitions

We evaluated in all patients the following anthropometric parameters: weight, height, and BMI. The weight (kilograms) and height (meters) were measured using a scale with an altimeter (Seca-, Hamburg, Germany). The BMI was calculated as “weight over height squared”.

According to BMI, patients were divided into the following categories: BMI < 18.5 kg/m^2^ underweight patients, BMI 18.5–24.9 kg/m^2^ normal weight patients, BMI 25–29.9 kg/m^2^ overweight patients, BMI ≥ 30 kg/m^2^ obese patients.

Radiological evaluation was performed at baseline (before treatment) and periodically (on average at 3, 6 months and thereafter annually) with computed tomography (CT) scan with contrast medium. Response to treatment was classified as Partial Response (PR), Stable Disease (SD), and Progressive Disease (PD) according to Response Evaluation Criteria in Solid Tumors (RECIST) v.1.1 [22,23]. The time from TKI administration to the first evidence of tumor progression or until death was defined as Progression-free survival (PFS). Overall Survival (OS) was considered as the time from the start date of the TKI treatment to the time of death from cancer disease. The sum of Target Lesions (TL) was calculated as the sum of diameters (longest for non-nodal lesions, short axis for nodal lesions) of all target lesions measured at baseline, according to RECIST criteria. 

**Figure 1:**
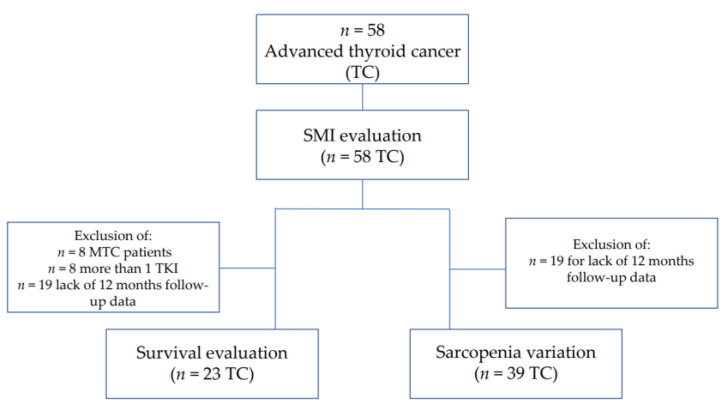
Flow chart.

The PFS and OS were evaluated in a subpopulation of 23 patients (Figure 1) all with DTC or PDTC and treated with only one TKI (lenvatinib or sorafenib), to avoid the potential bias due to different histotypes and multiple lines of TKI treatment.

A radiologist identified, for each patient, the single axial image at the level of the third lumbar vertebrae (L3) on which both transverse processes were fully observed, including psoas, erector spinae, quadratus lumborum, transversus abdominis, external and internal obliques, and rectus abdominis. Based on these images, was obtained the Skeletal Muscle Area (SMA; the cross-sectional area of all skeletal muscles at the L3 level). SMA was measured using the attenuation thresholds of −29 to +150 Hounsfield units. The Skeletal Muscle Index (SMI) was later calculated as SMA normalized by height squared and reported as cm^2^/m^2^.

The SMI cut-off to define sarcopenia was chosen among those proposed by the most recent European consensus [14] and it refers to a population of healthy subjects from the United States [24]. According to the SMI cut-off of 34.4 cm^2^/m^2^ for females and 45.4 cm^2^/m^2^ for males, patients were divided into a Sarcopenia Group (SG) and a Non-Sarcopenia Group (NSG). 

The percentage of loss of SMI (∆SMI%) was calculated as “(baseline SMI—SMI at time x)/SMI baseline × 100”, whereas the SMI, in addition to the baseline, was calculated at different times, including 3, 6, 12 months (time x) after the start of treatment. Skeletal Muscle Index and sarcopenia variations were assessed in a sub-group of 39 patients with a period of observation of at least 12 months with available clinical, biochemical, anthropometric, and radiological data at 3 and 12 months after starting TKI treatment (Figure 1).

In a subgroup of patients with available data, the Controlling Nutritional Status (CONUT) score was assessed at baseline (before starting TKI treatment) and during follow-up. It was defined as the sum of the following parameters, as described in the literature [25]: for serum albumin levels > 3.5, between 3.0 and 3.49, between 2.5 and 2.99 and <2.5 g/dL, 0, 2, 4, and 6 points were assigned, respectively; for serum total cholesterol levels > 180, between 140 and 179, between 100 and 139 and <100 mg/dL, 0, 1, 2, and 3 points were assigned, respectively; for serum total lymphocyte count > 1600, between 1200 and 1599, between 800 and 1199 and <800/mm^3^, 0, 1, 2, and 3 points were assigned, respectively.

The concentrations of albumin, total cholesterol, and total lymphocyte count were measured with standard colorimetric methods using the Cobas c 701/702 analyzer (Roche/Hitachi, Mannheim, Germany), obtained by fasting venous blood samples.

### 2.3. Statistical Analysis 

According to a preliminary descriptive analysis, quantitative variables were summarized by mean ± standard deviation, median and minimum-maximum range and qualitative variables by absolute frequencies and percentages. 

The comparison of the quantitative variables was performed by *t*-test and Mann–Whitney test, based on normality verified by the Kolmogorov–Smirnov test, while the Chi-square test and Fisher’s exact test were used to compare qualitative variables.

The repeated measures ANOVA was used to compare the value of SMI and ∆SMI% at different times (3, 6, and 12 months).

ROC (Receiver Operating Characteristic) curve analysis was performed to find the best SMI cut-off, in male and female patients, and the best BMI cut-off able to predict the presence of sarcopenia after 12 months of treatment. 

Progression Free Survival and Overall Survival were assessed by Kaplan–Meier and Cox regression. Hazard ratios and their 95% confidence interval (CI) were estimated. Stepwise Cox regression was performed, to identify factors contributing to Overall or Progression Free Survival. A *p*-value < 0.05 was considered statistically significant. The analyses were performed with SPSS statistics version 27.0 (IBM Corp, Armonk, NY, USA) and StatView version 5.0.1 for Windows (SAS Institute Inc., Cary, NC, USA). 

## 3. Results

### 3.1. Clinical-Pathological Features of the Whole Cohort of Patients 

A total of 28 (48.3%) patients were female and 30 (51.7%) were male. The mean age at the time of TKI treatment was 67.5 ± 13.8 years (median 69 years, 30–96 years). 

Histological diagnosis was DTC in 35 patients (60.4%), PDTC in 11 patients (18.9%), and MTC in 12 (20.7%). Moreover, 55 (94.8%) had distant metastasis (in particular, among them 25 patients had bone metastasis), and 3 patients (5.2%) had loco-regional metastasis. In detail, the number of anatomical sites involved by metastatic disease was one in 11 patients (18.9%), two in 15 patients (25.9%), three in 19 patients (32.8%), four in seven patients (12.1%) and five in six patients (10.3%). In 48 patients, it was possible to measure the baseline sum of the target lesion, resulting in 62.95 cm (mean 76.45 ± 47.88 cm; range 25–218).

The age at the start of TKI treatment ranged from 25 to 96 years (mean age 64 ± 16 years; median 67 years). The time lapse between cancer diagnosis and TKI treatment start ranged from 0.06 to 18.8 years (mean 6.3 ± 5.0, median 5.57 years); the median TKI therapy duration was 25.9 months (range 0.06–131).

The TKI used as first-line therapy was lenvatinib in 22 patients (37.93%), sorafenib in 22 patients (37.93%), vandetanib in 10 patients (17.24%), motesanib in three patients (5.17%) and cabozantinib in one patient (1.72%). Out of the 58 patients, 19 (32.75%) were treated with other TKIs.

The median pre-treatment weight was 74.7 kg (mean 75.9 ± 17.3; range 44–131), and the median body mass index (BMI) at baseline was 26 kg/m^2^ (mean 26 ± 5.9; range 18–46.9). Particularly, only one patient (1.72%) resulted as underweight before TKI treatment start, 21 patients (36.2%) were of normal weight, 19 patients (32.76%) were overweight, and the remaining 17 (29.31%) were obese patients. 

The performance status at baseline, according to the Eastern Cooperative Oncology Group (ECOG) scale, was 0 in 50 patients (86.2%), one in six patients (10.3%), and two in two patients (3.5%).

Controlling Nutritional Status (CONUT) score was 0–1 (absence of malnutrition) in 19/42 patients (45.2%), 2–4 (presence of light malnutrition) in 18/42 patients (42.9%), 5–7 (presence of mild malnutrition) in 5/42 patients (11.9%). No patient had severe malnutrition (CONUT score 9–12).

### 3.2. Sarcopenia before Starting TKI Treatment

The median SMI value before TKI treatment was 46.1 cm^2^/m^2^ (mean 48 ± 10.4; range 28.6–78.4). According to the SMI cut-off of 34.4 cm^2^/m^2^ for women and 45.4 cm^2^/m^2^ for men [24], patients were divided into SG and NSG (Table 1). In our cohort, baseline sarcopenia was recorded in 12/58 patients (20.69%). 

No significant differences between the two groups for clinical-pathological data collected were observed, except for SMI value (38.85 cm^2^/m^2^ in SG vs. 49.6 cm^2^/m^2^ in NSG, *p* = 0.0002), BMI (median 23.39 kg/m^2^ in SG vs. 26.94 kg/m^2^ in NSG, *p* = 0.004), and BMI categories (*p* = 0.01).

### 3.3. Baseline Sarcopenia and Response to TKI Treatment

At the last follow-up, 14 patients (24.14%) were under TKI treatment, among them 10 with the first line therapy (9/10 patients with lenvatinib and 1/10 patients with vandetanib), four patients (6.9%) were lost at follow up, 39 patients (67.24%) died due to thyroid disease or for other reasons, and anticancer treatment was withdrawn in one patient (1.72%). The duration of the first TKI treatment was 30.0 months (median 25.9 months, range 0.9–131). The duration of the first TKI treatment was significantly shorter in patients with sarcopenia than in non-sarcopenic patients (19.1 months vs. 28.78 months, *p* = 0.012). 

Median PFS was 18.1 months and it resulted as significantly longer in non-sarcopenic (24.39 ± 18.96 months) than in sarcopenic (8.46 ± 6.87 months) patients (*p* = 0.008). Analyzing OS, according to the presence or the absence of pre-treatment sarcopenia, we found better survival in non-sarcopenic patients, approaching the borderline of significance (53.2 ± 41.8 months vs. 37.1 ± 44.3 months, *p* = 0.07). 

Stepwise Cox regression was used to analyze factors contributing to PFS and OS. Variables included in the analysis were age at the beginning of the first TKI, BMI categories (obesity plus over-weight vs. normal weight), pre-treatment weight and SMI, gender, pre-treatment sarcopenia (present vs. absent), number of sites of distant metastases, the sum of the target lesions diameters, ECOG performance status (0 vs. ≥1), and the number of TKI line of treatment (>1 TKI vs. 1 TKI). We found that older age at the beginning of the first TKI (HR 1.05; 95% CI, 1.02–1.09, *p* = 0.006), presence of pre-treatment sarcopenia (HR 4.29; 95% CI, 1.21–15.11, *p* = 0.02), higher number of sites of distant metastases (HR 1.55; 95% CI, 1.09–2.2, *p* = 0.014), higher sum of diameters of TL (HR 1.01; 95% CI, 1.002–1.019, *p* = 0.014), and treatment with more than one TKI (HR 3.10; 95% CI, 1.23–7.78, *p* = 0.016) negatively affected PFS (Table 2). The parameters that correlated with a poorer OS were older age at the beginning of the first TKI (HR 1.06; 95% CI, 1.03–1.09, *p* < 0.001), higher number of sites of distant metastases (HR 1.41; 95% CI, 1.01–1.96, *p* = 0.044), and a higher sum of diameters of TL (HR 1.01; 95% CI, 1.002–1.02, *p* = 0.012) (Table 2).

### 3.4. Sarcopenia Prevalence Variation during TKI Treatment

**Figure 2:**
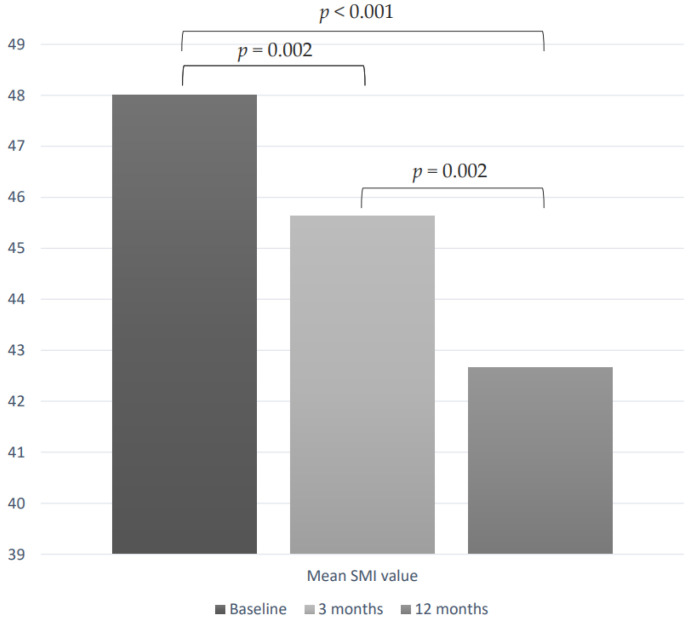
SMI values variations during TKI treatment in a subgroup of 39 patients.

As shown in Figure 1, in a subgroup of 39 patients with at least 12-month follow-up data, we evaluated SMI changes during the first line of TKI therapy. At the time of enrollment, the mean SMI value was 48.0 cm^2^/m^2^ (± 10.4). During TKI treatment, we observed a significant reduction of SMI values, which resulted as 45.6 cm^2^/m^2^ (± 9.4) after 3 months (*p* = 0.002 vs. baseline) and 42.6 cm^2^/m^2^ (±10.9) after 12 months of therapy (*p* < 0.0001 vs. baseline and *p* = 0.002 vs. 3 months) (Figure 2). After 3 and 12 months of treatment, the mean ∆SMI% was −4.4% (±7.7) and −10.6% (±8.3), respectively. 

At the 12-month follow-up, all sarcopenic patients at baseline (*n* = 6 patients) resulted in remaining sarcopenic. Among 33 non-sarcopenic patients, 9/33 (27.3%) developed a sarcopenic condition after 12 months of TKI treatment. Overall, at the 12-month follow-up, 45.4% of patients were sarcopenic. This rate was 33% in the subgroup of DTC patients treated with lenvatinib.

### 3.5. Prognostic Factors of Sarcopenia Development during TKI Treatment

Among non-sarcopenic patients at baseline, we evaluated prognostic factors for the development of sarcopenia during TKI treatment (Table 3). 

SMI value at baseline was significantly lower in patients who developed sarcopenia compared with those without sarcopenia at 12 months follow-up (42.7 cm^2^/m^2^ vs. 51.8 cm^2^/m^2^, *p* = 0.003). Similarly, weight (65.9 kg vs. 81.4 kg, *p* = 0.04) and BMI (24.5 kg/m^2^ vs. 30.1 kg/m^2^, *p* = 0.02) were lower in patients who developed sarcopenia. Sarcopenia at the 12-month follow-up was more frequent in patients with bone metastases (50% of cases vs. 14% of cases in patients without bone metastases, *p* = 0.02). The development of sarcopenia at 12 months of treatment did not correlate with age, gender, ECOG Performance Status, histology, type of TKI used as first line, number of sites of distant metastases, the sum of diameters of target lesion, the time from cancer diagnosis and TKI start, duration of treatment with first TKI, the best response with the first TKI therapy and the number of TKI treatment lines.

At multivariate analysis, the only parameter that correlated with sarcopenia development at 12 months follow-up was the SMI value at baseline (OR 0.89, CI 0.78–0.98, *p* = 0.04).

**Figure 3:**
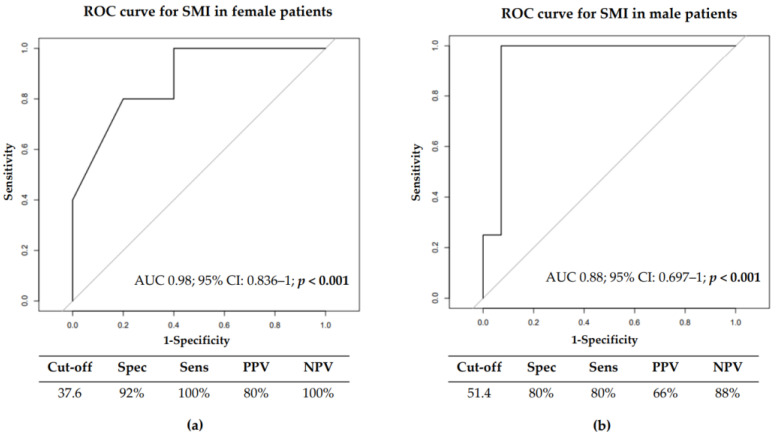
ROC curve for Skeletal Muscle Index in female (**a**) and male (**b**) in 33 non-sarcopenic patients at baseline.

We also investigated the best SMI cut-off able to predict sarcopenia development by ROC curves analysis. The best SMI cut-off for the female population was 37.6 cm^2^/m^2^. This cut-off had a specificity of 92% and a sensitivity of 100%, with an Area Under the Curve (AUC) of 0.940 95% CI: 0.8358–1 (*p* < 0.0001) (Figure 3a). In male patients, the SMI cut-off identified was 51.4 cm^2^/m^2^, with a specificity and sensitivity of 80%, with an AUC of 0.88, 95% CI: 0.697–1 (*p* < 0.0001) (Figure 3b). 

## 4. Discussion

Sarcopenia is a frequent condition in oncology with a prevalence of 35.3% [26]. This prevalence is higher in palliative settings vs. curative settings and it also results differently in various tumors [26]. Sarcopenia has been extensively reported as a prognostic factor for several human cancers [3,11,12,13]. Recent studies have shown that a lower SMI value at cancer diagnosis is associated with poor survival in patients with solid tumors [17]. It has also been demonstrated that sarcopenia is associated with a higher incidence of anti-cancer drug toxicities and with a higher risk of post-surgical complications in oncological patients [16]. 

Nevertheless, data regarding the prevalence as well as the association between sarcopenia and response to TKI treatment in patients with advanced thyroid cancer, are still lacking. The first set of data was derived from a retrospective study of the Phase III DECISION and ZETA trials. The prevalence of sarcopenia was 49.9%, with a higher prevalence in Asian subjects (73.3%) than in Europeans (32.2) [18]. In two recent Japanese studies, the presence of pre-treatment sarcopenia, measured by SMI, was 61% and 39.1%, respectively [20,21]. 

In our cohort of 58 Caucasian thyroid cancer patients, sarcopenia was found in about 20% of them before starting TKI treatment. This rate was lower than that observed in other studies on thyroid cancer patients in which the prevalence of sarcopenia ranged from 39% to 61.1% [18,20,21]. A possible explanation of these findings might be the differences in the thyroid cancer population included in the studies. Indeed, in the study of Nishiyama et al. [21], thyroid cancer patients were prior treated with chemotherapy in 13% of cases, the ECOG score 3–4 was present in about 20% of patients and a very high sum of lesions was reported, suggesting more advanced disease. Conversely, in our study, none of the thyroid cancer patients were treated with chemotherapy, the ECOG score ranged from 0 to 2 and all patients were treatment-naive with TKI. In addition, such differences in the prevalence of sarcopenia may also be due to the differences in ethnicities (Caucasian vs. Asian population) between our study and the two published ones [20,21]. Indeed, as previously reported, the rate of sarcopenia was greater in Asian than in European thyroid cancer patients [18]. 

Several factors contribute to sarcopenia onset in cancer patients. The pathophysiology of cancer sarcopenia is characterized by a negative protein and energy balance, driven by a variable combination of abnormal metabolism and reduced food intake [27]. Tumor-associated inflammation induces alterations in metabolism and cell apoptosis of the skeletal musculature due to pro-inflammatory cytokines [10,28]. Moreover, in these patients, long-term thyroxine suppressive therapy may also contribute [18]. The side effects of TKI such as diarrhea, nausea, vomiting, mucositis, xerostomia, and dysgeusia might further reduce food intake, leading to a condition of sarcopenia [29,30]. Finally, the intrinsic mechanism of TKI itself could contribute to the development of this condition [31].

In our study, sarcopenia correlated with response to TKI therapy. Particularly, the duration of TKI treatment was shorter, with a PFS significantly poorer in sarcopenic compared to non-sarcopenic patients. The role of sarcopenia in advanced thyroid cancer patients was also confirmed with Stepwise Cox regression analysis. Pre-treatment sarcopenia negatively affected PFS in patients with advanced thyroid, suggesting that sarcopenia might be a negative prognostic factor for TKI treatment. 

Conversely, although we found better survival in non-sarcopenic patients, approaching the borderline of significance (53.2 ± 41.8 months vs. 37.1 ± 44.3 months, *p* = 0.07), in Cox regression analysis, pre-treatment sarcopenia was not found to be an independent risk factor for OS, probably due to the small cohort of patients. 

Our results are comparable to those reported in an Asian population with advanced thyroid cancer, in which the presence of pre-treatment sarcopenia, measured by SMI, led to a poor outcome [20,21]. Yamazaki and colleagues [20] evaluated 54 patients with DTC and MTC, treated with lenvatinib or vandetanib, demonstrating that sarcopenic patients had a poorer PFS compared to non-sarcopenic patients (*p* = 0.017). In this study, pre-treatment sarcopenia was the only independent prognostic factor for PFS. Nishiyama and colleagues [21] also found a worse prognosis in sarcopenic than in non-sarcopenic patients.

Only a few studies have investigated the effects of TKI treatment both on anthropometric parameters and body composition [18,19,32]. Huillard and colleagues demonstrated that sorafenib treatment significantly reduced lean body mass (measured at L3). In our study, a significant reduction of SMI values was observed in a subgroup of advanced thyroid cancer patients after 3 months (−4.4%) and 12 months (−10.6%) of TKI therapy. Furthermore, 27% of non-sarcopenic patients at baseline developed a sarcopenic condition after 12 months of TKI treatment. Overall, in our cohort, at the end of a period of about one year of observation, sarcopenia prevalence was around 40% (vs 20.7% at baseline). Similarly, a significant decrease in lean body mass was observed in patients receiving sorafenib, suggesting a significant effect of TKI on muscle mass [18]. Unlike our study, the observation period was only 6 months. It was not well defined if this progressive decrease of lean body mass during TKI treatment may be due to the therapy related-side effects or to a worsening of tumor-associated inflammation. 

The only independent prognostic factor of sarcopenia development during TKI treatment was the SMI value at baseline (OR 0.89, CI 0.78–0.98, *p* = 0.04). Using ROC curve analysis, we identified SMI cut-offs able to predict the evolution towards sarcopenia. Specifically, using a SMI cut-off of 37.6 cm^2^/m^2^ for females and 51.4 cm^2^/m^2^ for males, we were able to identify patients who developed sarcopenia during TKI treatment with a sensitivity and specificity of more than 80%. 

Some limitations related to this study include its retrospective design, the relatively limited sample size, the use of different TKIs, and the partial data regarding drug dosage and adverse events during TKI treatment. Moreover, additional tests able to evaluate the physical performance of patients were not available and the SMI cut-off was calculated using a small cohort of patients without sample size calculation. Nevertheless, this study yielded significant results, since, to our knowledge, this is the first study that has demonstrated that TKI therapy leads to a significant loss of muscle mass, measured by SMI, in thyroid cancer patients. It is also the first study to demonstrate the role of pre-treatment sarcopenia as a prognostic factor in a Caucasian population of patients with thyroid carcinoma.

## 5. Conclusions

Sarcopenia may be a useful outcome predictor for advanced thyroid cancer patients undergoing TKI treatment. This study highlights the importance of assessing a condition of sarcopenia, both before and during TKI therapy, because prognosis seems to be affected by muscle loss. Further, sarcopenia could potentially be the target of treatment interventions to improve the prognosis in such populations. This evidence further supports the need for a multidisciplinary approach in advanced thyroid cancer [33] that also includes the prevention and treatment of malnutrition and sarcopenia in adult thyroid cancer patients [34].

The prevention of this condition, for example by establishing personalized nutritional support or physical activity programs, ideally before starting anticancer treatment, could be the strategy to obtain a better efficacy of therapy. However, prospective or interventional clinical studies with a larger sample size are needed to validate the correlation between muscle mass loss during TKI therapy and treatment outcomes in patients with advanced thyroid cancer.

## Figures and Tables

**Table 1:** Clinical-pathological features of the whole cohort of patients according to the presence/absence of sarcopenia before TKI treatment.

	All patients (*n* = 58)	Sarcopenia Group (*n* = 12)	Non-Sarcopenia Group (*n* = 46)	*p* Value
**Age**, years				0.51
Median (range)	67 (25–96)	64.97 (34.3–87.1)	67.13 (25.2–96.2)	
Mean (SD)	64 (15.9)	66.8 (16.12)	63.34 (15.97)	
**Gender**, *n* (%)				0.33
Female	28 (48.2%)	4 (33.3%)	24 (52.2%)	
Male	30 (51.8%)	8 (66.7%)	22 (47.8%)	
**ECOG PS**, *n* (%)				0.29
0	50 (86.2%)	12 (100%)	38 (82.6%)	
1	6 (10.3%)	0 (0%)	6 (13%)	
2	2 (3.5%)	0 (0%)	2 (4.4%)	
**Histology**, *n* (%)				0.73
DTC	35 (60.34%)	6 (50%)	29 (63.04%)	
PDTC	11 (18.96%)	3 (25%)	8 (17.39%)	
MTC	12 (20.68%)	3 (25%)	9 (19.56%)	
**Weight**, kg				0.052
Median (range)	74.7 (44–131)	70.5 (44–89.5)	76.5 (47.8–131)	
Mean (SD)	75.9 (17.3)	68.58 (12.75)	77.86 (18)	
**BMI**, kg/m^2^				**0.004**
Median (range)	26.0 (18–46.9)	23.4 (18.1–31.1)	26.9 (18.7–46.9)	
Mean (SD)	26 (5.9)	23.86 (3.7)	28.2 (6.08)	
**BMI categories**, *n* (%)				**0.01**
<18.5 kg/m^2^	1 (1.72%)	1 (8.33%)	0 (0%)	
18.5–24.9 kg/m^2^	21 (36.20%)	8 (66.67%)	13 (28.26%)	
25–29.9 kg/m^2^	19 (32.76%)	2 (16.67%)	17 (36.95%)	
≥30 kg/m^2^	17 (29.31%)	1 (8.33%)	16 (34.78%)	
CONUT score *				0.49
0–1	19 (45.2%)	3 (30%)	16 (48.5%)	
2–4	18 (42.9%)	4 (40%)	14 (42.4%)	
5–7	5 (11.9%)	3 (30%)	3 (9.1%)	
**SMI value**, cm^2^/m^2^				**0.0002**
Median (range)	46.1 (28.6–78.4)	38.85 (28.6–45.3)	49.6 (32.5–78.4)	
Mean (SD)	48.0 (10.4)	38.9 (5.65)	50.3 (10.1)	
**Number of anatomical sites involved** *n* (%)				0.48
1	11 (18.96%)	3 (25%)	8 (17.39%)	
2	15 (25.86%)	1 (8.33%)	14 (30.43%)	
≥3	32 (55.18%)	8 (66.67%)	24 (52.17%)	
**Number of patients with bone metastasis**, *n* (%)	25 (43%)	8 (66.7%)	17 (36.9%)	0.10
Sum of Target Lesions **, cm				0.83
Median (range)	62.95 (25–218)	60 (25–128)	65 (26–192)	
Mean (SD)	76.45 (47.88)	81.22 (62.78)	75.34 (44.72)	
**Time since diagnosis**, months				0.29
Median (range)	5.57 (0.06–18.8)	7.99 (0.80–13.87)	5.36 (0.06–18.8)	
Mean (SD)	6.38 (5.04)	7.76 (4.46)	6.02 (5.17)	

* Evaluated in 42 patients; ** Evaluated in 48 patients.

**Table 2:** Stepwise Cox regression for PFS and OS.

	Progression Free Survival		Overall Survival	
	HR (95% CI)	*p*	HR (95% CI)	*p*
**Age at TKI start**	1.05 (1.02–1.09)	0.006	1.06 (1.03–1.09)	< 0.001
**Sarcopenia** Yes vs. No	4.29 (1.21–15.11)	0.02	-	-
**N. of anatomical site involved**	1.55 (1.09–2.20)	0.014	1.41 (1.01–1.96)	0.044
**Sum of Target Lesions diameters**	1.01 (1.002–1.019)	0.014	1.01 (1.002–1.02)	0.012
**Number of TKI treatment** >1 vs. 1	3.10 (1.23–7.78)	0.016	-	-

**Table 3:** Clinical-pathological features of 33 non-sarcopenic patients at baseline according to the development of sarcopenia after 12 months of treatment.

	All Patients (*n* = 33)	Sarcopenia Group (*n* = 9)	Non-Sarcopenia Group (*n* = 24)	*p* Value
**Age at TKI start**, years				0.51
Median (range)	67.1 (25.2–96.2)	72 (25.2–79.8)	65.8 (30.0–96.2)	
Mean (SD)	63.3 (15.9)	63.9 (18.0)	62.7 (16.2)	
**Gender**, *n* (%)				0.47
Female	18 (54.5%)	4 (22.2%)	14 (77.8%)	
Male	15 (45.5%)	5 (33.3%)	10 (66.7%)	
**ECOG PS**, *n* (%)				0.66
0	27 (81.8%)	7 (25.9%)	20 (74.1%)	
1	5 (15.2%)	2 (40.0%)	3 (60.0%)	
2	1 (3%)	0 (0.0%)	1 (100.0%)	
**Histology**, *n* (%)				0.67
DTC	19 (57.6%)	6 (31.6%)	13 (68.4%)	
PDTC	7 (21.2%)	2 (28.6%)	5 (71.4%)	
MTC	7 (21.2%)	1 (14.3%)	6 (85.7%)	
**Weight**, kg				**0.04**
Median (range)	76.5 (47.8–131)	67 (50–87)	83 (47.8–131)	
Mean (SD)	77.9 (18)	65.9 (11.98)	81.4 (21.15)	
**BMI**, kg/m^2^				**0.02**
Median (range)	26.9 (18.7–46.9)	24.5 (18.7–30.8)	28.7 (20.6–46.9)	
Mean (SD)	28.2 (6.1)	24.5 (3.7)	30.1 (7.1)	
**SMI value**, cm^2^/m^2^				**0.003**
Median (range)	49.6 (32.5–78.4)	45.8 (32.5–53)	49.7 (34.5–78.4)	
Mean (SD)	50.4 (10.1)	42.7 (7.7)	51.8 (10.8)	
**Number of anatomical sites involved** *n* (%)				0.14
1	5 (15.2%)	1 (20.0%)	4 (80.0%)	
2	10 (30.3%)	1 (10.0%)	9 (90.0%)	
≥3	18 (54.5%)	7 (38.9%)	11 (61.1%)	
**Bone metastasis**, *n* (%)				**0.02**
Yes	12 (36.4%)	6 (50.0%)	6 (50.0%)	
No	21 (63.6%)	3 (14.0%)	18 (76%)	
**TKI**, *n* (%)				0.8
Lenvatinib	12 (36.3%)	3 (25%)	9 (75%)	
Others TKI *	21 (63.7%)	6 (28.6%)	15 (71.4%)	
**Sum of Target Lesions** **, cm				0.17
Median, range)	65 (26–192)	75 (35–192)	50 (26–171)	
Mean (SD)	75.3 (44.7)	92.2 (54.6)	70.8 (48.1)	
**Time since diagnosis**, years				0.77
Median (range)	5.4 (0.07–18.8)	4.7 (0.5–9.6)	5.5 (0.1–18.8)	
Mean (SD)	6 (5.17)	5.1 (3.42)	6.6 (5.84)	
**Duration of treatment with first TKI**, months				0.29
Median (range)	27.5 (0.9–131)	32.1 (9.9–57.1)	36 (10–131)	
Mean (SD)	33.5 (26.6)	30.4 (16.78)	44.2 (30.3)	
**Best Response**				0.64
PD	2 (6.1%)	0 (0.0%)	2 (100.0%)	
PR	15 (45.4%)	4 (26.7%)	11 (73.3%)	
SD	16 (48.5%)	5 (31.2%)	11 (68.8%)	
**Number of treatment lines**, *n* (%)				0.27
Only 1 TKI	23 (69.7%)	5 (21.7%)	18 (78.3%)	
More than 1 TKI	10 (30.3%)	4 (40.0%)	6 (60.0%)	

* Sorafenib, Vandetanib, Motesanib; ** Data available in nine non-sarcopenic and 23 sarcopenic patients.

## Data Availability

Data are contained within the article.

## References

[1] Meza-Valderrama D., Marco E., Dávalos-Yerovi V., Muns M.D., Tejero-Sánchez M., Duarte E., Sánchez-Rodríguez D. (2021). Sarcopenia, Malnutrition, and Cachexia: Adapting Definitions and Terminology of Nutritional Disorders in Older People with Cancer. Nutrients.

[2] Gingrich A., Volkert D., Kiesswetter E., Thomanek M., Bach S., Sieber C.C., Zopf Y. (2019). Prevalence and overlap of sarcopenia, frailty, cachexia and malnutrition in older medical inpatients. BMC Geriatr..

[3] Shachar S.S., Williams G.R., Muss H.B., Nishijima T.F. (2016). Prognostic value of sarcopenia in adults with solid tumours: A meta-analysis and systematic review. Eur. J. Cancer.

[4] Jain R., Handorf E., Khare V., Blau M., Chertock Y., Hall M.J. (2020). Impact of Baseline Nutrition and Exercise Status on Toxicity and Outcomes in Phase I and II Oncology Clinical Trial Participants. Oncologist.

[5] Bullock A.F., Greenley S.L., McKenzie G.A.G., Paton L.W., Johnson M.J. (2020). Relationship between markers of malnutrition and clinical outcomes in older adults with cancer: Systematic review, narrative synthesis and meta-analysis. Eur. J. Clin. Nutr..

[6] Cederholm T., Barazzoni R., Austin P., Ballmer P., Biolo G., Bischoff S.C., Compher C., Correia I., Higashiguchi T., Holst M. (2017). ESPEN guidelines on definitions and terminology of clinical nutrition. Clin. Nutr..

[7] Fearon K., Strasser F., Anker S.D., Bosaeus I., Bruera E., Fainsinger R.L., Jatoi A., Loprinzi C., MacDonald N., Mantovani G. (2011). Definition and classification of cancer cachexia: An international consensus. Lancet Oncol..

[8] Evans W.J., Morley J.E., Argilés J., Bales C., Baracos V., Guttridge D., Jatoi A., Kalantar-Zadeh K., Lochs H., Mantovani G. (2008). Cachexia: A new definition. Clin. Nutr..

[9] Muscaritoli M., Anker S.D., Argilés J., Aversa Z., Bauer J.M., Biolo G., Boirie Y., Bosaeus I., Cederholm T., Costelli P. (2010). Consensus definition of sarcopenia, cachexia and pre-cachexia: Joint document elaborated by Special Interest Groups (SIG) “cachexia-anorexia in chronic wasting diseases” and “nutrition in geriatrics”. Clin. Nutr..

[10] Cruz-Jentoft A.J., Baeyens J.P., Bauer J.M., Boirie Y., Cederholm T., Landi F., Martin F.C., Michel J.P., Rolland Y., Schneider S.M. (2010). European Working Group on Sarcopenia in Older People. Sarcopenia: European consensus on definition and diagnosis: Report of the European Working Group on Sarcopenia in Older People. Age Ageing.

[11] Park S.E., Hwang I.G., Cho C.H., Kang H., Kim B.G., Park B.K., Cha S.J., Jang J.S., Choi J.H. (2018). Sarcopenia is poor prognostic factor in older patients with locally advanced rectal cancer who received preoperative or postoperative chemoradiotherapy. Medicine.

[12] Ota T., Ishikawa T., Endo Y., Matsumura S., Yoshida J., Yasuda T., Okayama T., Inoue K., Dohi O., Yoshida N. (2019). Skeletal muscle mass as a predictor of the response to neoadjuvant chemotherapy in locally advanced esophageal cancer. Med. Oncol..

[13] Shiroyama T., Nagatomo I., Koyama S., Hirata H., Nishida S., Miyake K., Fukushima K., Shirai Y., Mitsui Y., Takata S. (2019). Impact of sarcopenia in patients with advanced nonsmall cell lung cancer treated with PD-1 inhibitors: A preliminary retrospective study. Sci. Rep..

[14] Cruz-Jentoft A.J., Bahat G., Bauer J., Boirie Y., Bruyère O., Cederholm T., Cooper C., Landi F., Rolland Y., Sayer A.A. (2019). Writing Group for the European Working Group on Sarcopenia in Older People 2 (EWGSOP2), and the Extended Group for EWGSOP2 (2019). Sarcopenia: Revised European consensus on definition and diagnosis. Age Ageing.

[15] Shen W., Punyanitya M., Wang Z., Gallagher D., St-Onge M.P., Albu J., Heymsfield S.B., Heshka S. (2004). Total body skeletal muscle and adipose tissue volumes: Estimation from a single abdominal cross-sectional image. J. Appl. Physiol..

[16] Pamoukdjian F., Bouillet T., Lévy V., Soussan M., Zelek L., Paillaud E. (2018). Prevalence and predictive value of pre-therapeutic sarcopenia in cancer patients: A systematic review. Clin Nutr..

[17] Rinninella E., Cintoni M., Raoul P., Ponziani F.R., Pompili M., Pozzo C., Strippoli A., Bria E., Tortora G., Gasbarrini A. (2021). Prognostic value of skeletal muscle mass during tyrosine kinase inhibitor (TKI) therapy in cancer patients: A systematic review and meta-analysis. Intern Emerg. Med..

[18] Huillard O., Jouinot A., Tlemsani C., Brose M.S., Arrondeau J., Meinhardt G., Fellous M., De Sanctis Y., Schlumberger M., Goldwasser F. (2019). Body Composition in Patients with Radioactive Iodine-Refractory, Advanced Differentiated Thyroid Cancer Treated with Sorafenib or Placebo: A Retrospective Analysis of the Phase III DECISION Trial. Thyroid.

[19] Massicotte M.H., Borget I., Broutin S., Baracos V.E., Leboulleux S., Baudin E., Paci A., Deroussent A., Schlumberger M., Antoun S. (2013). Body composition variation and impact of low skeletal muscle mass in patients with advanced medullary thyroid carcinoma treated with vandetanib: Results from a placebo-controlled study. J. Clin. Endocrinol. Metab..

[20] Yamazaki H., Sugino K., Matsuzu K., Masaki C., Akaishi J., Hames K., Tomoda C., Suzuki A., Uruno T., Ohkuwa K. (2020). Sarcopenia is a prognostic factor for TKIs in metastatic thyroid carcinomas. Endocrine.

[21] Nishiyama A., Staub Y., Suga Y., Fujita M., Tanimoto A., Ohtsubo K., Yano S. (2021). Sarcopenia may Influence the Prognosis in Advanced Thyroid Cancer Patients Treated With Molecular Targeted Therapy. In Vivo.

[22] Eisenhauer E.A., Therasse P., Bogaerts J., Schwartz L.H., Sargent D., Ford R., Dancey J., Arbuck S., Gwyther S., Mooney M. (2009). New response evaluation criteria in solid tumours: Revised RECIST guideline (version 1.1). Eur. J. Cancer.

[23] Schwartz L.H., Litière S., de Vries E., Ford R., Gwyther S., Mandrekar S., Shankar L., Bogaerts J., Chen A., Dancey J. (2016). RECIST 1.1-Update and clarification: From the RECIST committee. Eur. J. Cancer.

[24] Derstine B.A., Holcombe S.A., Ross B.E., Wang N.C., Su G.L., Wang S.C. (2018). Skeletal muscle cutoff values for sarcopenia diagnosis using T10 to L5 measurements in a healthy US population. Sci. Rep..

[25] Ignacio de Ulíbarri J., González-Madroño A., De Villar N.G., González P., Gonzalez B., Mancha A., Rodriguez F., Fernandez G. (2005). CONUT: A tool for Controlling Nutritional Status. First validation in a hospital population. Nutr. Hosp..

[26] Surov A., Wienke A. (2022). Prevalence of sarcopenia in patients with solid tumors: A meta-analysis based on 81,814 patients. JPEN J. Parenter Enteral Nutr..

[27] Fearon K.C., Glass D.J., Guttridge D.C. (2012). Cancer cachexia: Mediators, signaling, and metabolic pathways. Cell Metab..

[28] Roubenoff R., Parise H., Payette H.A., Abad L.W., D’Agostino R., Jacques P.F., Wilson P.W., Dinarello C.A., Harris T.B. (2003). Cytokines, insulin-like growth factor 1, sarcopenia, and mortality in very old community-dwelling men and women: The Framingham Heart Study. Am. J. Med..

[29] Cabanillas M.E., Takahashi S. (2019). Managing the adverse events associated with lenvatinib therapy in radioiodine-refractory differentiated thyroid cancer. Semin Oncol..

[30] Krajewska J., Paliczka-Cieslik E., Jarzab B. (2017). Managing tyrosine kinase inhibitors side effects in thyroid cancer. Expert Rev. Endocrinol. Metab..

[31] Rinninella E., Cintoni M., Raoul P., Pozzo C., Strippoli A., Ponziani F.R., Pompili M., Bria E., Tortora G., Gasbarrini A. (2020). Skeletal Muscle Loss during Multikinase Inhibitors Therapy: Molecular Pathways, Clinical Implications, and Nutritional Challenges. Nutrients.

[32] De Leo S., Colombo C., Di Stefano M., Dubini A., Cozzi S., Persani L., Fugazzola L. (2020). Body Composition and Leptin/Ghrelin Levels during Lenvatinib for Thyroid Cancer. Eur. Thyroid J..

[33] Giovanella L., Scappaticcio L. (2019). Radioiodine therapy of advanced differentiated thyroid cancer: Clinical considerations and multidisciplinary approach. Q. J. Nucl. Med. Mol. Imaging.

[34] Arends J., Bachmann P., Baracos V., Barthelemy N., Bertz H., Bozzetti F., Fearon K., Hütterer E., Isenring E., Kaasa S. (2017). ESPEN guidelines on nutrition in cancer patients. Clin. Nutr..

